# Region Evolution eXplorer – A tool for discovering evolution trends in ontology regions

**DOI:** 10.1186/s13326-015-0020-6

**Published:** 2015-06-01

**Authors:** Victor Christen, Michael Hartung, Anika Groß

**Affiliations:** Department of Computer Science, Universität Leipzig, Augustusplatz 10, Leipzig, Germany; Interdisciplinary Center for Bioinformatics, Universität Leipzig, Härtelstr. 16 - 18, Leipzig, Germany

**Keywords:** Ontology evolution, Ontology visualization, Ontologies

## Abstract

**Background:**

A large number of life science ontologies has been developed to support different application scenarios such as gene annotation or functional analysis. The continuous accumulation of new insights and knowledge affects specific portions in ontologies and thus leads to their adaptation. Therefore, it is valuable to study which ontology parts have been extensively modified or remained unchanged. Users can monitor the evolution of an ontology to improve its further development or apply the knowledge in their applications.

**Results:**

Here we present REX (Region Evolution eXplorer) a web-based system for exploring the evolution of ontology parts (regions). REX provides an analysis platform for currently about 1,000 versions of 16 well-known life science ontologies. Interactive workflows allow an explorative analysis of changing ontology regions and can be used to study evolution trends for long-term periods.

**Conclusion:**

REX is a web application providing an interactive and user-friendly interface to identify (un)stable regions in large life science ontologies. It is available at http://www.izbi.de/rex.

**Electronic supplementary material:**

The online version of this article (doi:10.1186/s13326-015-0020-6) contains supplementary material, which is available to authorized users.

## Background

In recent years ontologies have become increasingly important for annotating, sharing and analyzing data in the life sciences [1,2]. For instance, functional term enrichment analysis [3] use ontologies to propagate information along their structure to find over-represented terms w.r.t. a list of interesting genes. The heavy usage of ontologies leads to a steady modification of their content [4,5]. In particular, ontologies are adapted to incorporate new knowledge, eliminate initial design errors or achieve changed requirements. Tools like Protégé [6] support the development and change of ontologies. This process is usually distributed since especially large ontologies can not be maintained by single developers, such that collaborative work is performed [6,7]. Typically, the overall development of an ontology is coordinated by a project leader or consortium, and multiple developers contribute knowledge in their field of expertise. Ontology providers release new versions on a regular basis or whenever a significant amount of changes were performed. Users should thus always consider the newest ontology version in their applications to avoid errors from previous versions and to be up-to-date w.r.t. the modeled knowledge.

Due to the ontology’s size and complexity, the problem arises that coordinators, developers and users want to know whether specific parts (regions) of a large ontology have changed or not. We see different use cases where a tool support is required:

- **Region Evolution Analysis**: Users may question which regions have evolved in what way in a specific period of time. For instance, there can be regions exhibiting a high degree of instability. These regions may have been in the focus of development and underlay many modifications. This might be caused by the topics modeled within these regions, e.g., current topics require permanent modifications to be up-to-date. By contrast, a stable region might be already completed or was of low interest during recent ontology development. Furthermore, interesting insights come up when studying the evolution of a region over time, e.g., by considering the change intensity in the past five years. Another use case would be the comparison of the evolution in different regions, e.g., a head-to-head comparison of two regions can provide information whether these regions have evolved in a similar way or show a different evolution behavior.
- **Ontology Development and Project Coordination**: In ontology development projects coordinators usually face the problem how to track and measure the ongoing development in an ontology. This especially holds for large and distributed projects when the ontology to be developed covers a number of different topics. In such cases project coordinators are interested in the evolution of different ontology parts. In particular, they like to see (1) how work has progressed and (2) like to detect potential for future development. Having a tool that can flexibly compute where, when and how many changes occurred, an improved project controlling and decision management can be achieved. For instance, if work in an area did not progress as planned, resources can be re-scheduled accordingly in order to complete the work.
  The controlling is not limited to project coordinators. Also, developers can inform themselves about the evolution in different regions and may find interesting starting points to participate, e.g., regions with topics they are aware of.
- **Dependent Data and Algorithms**: Biomedical datasets like genes, images or electronic health records are typically annotated with concepts of ontologies. Thus, they depend on the ontology content and exhibit another use case for REX. For instance, if a user considers the anatomy part of the NCI Thesaurus (NCIT) [8] for annotating local data such as radiology pictures, she would like to know how this part has evolved recently, i.e., is the part unstable or stable. Thus, one can estimate whether or not an adaptation of the annotations would be feasible. Moreover, ontology-based algorithms or applications might be affected by ontology changes. For instance, if results of a gene set enrichment analysis [3] are located in a strongly evolving ontology part, it should be re-done based on the newest ontology version to see how results change. By contrast, results located within stable ontology parts are likely to remain unchanged. In own previous work [9] we already used such techniques to figure out how the results of real gene set enrichment analyses changed over time and how these changes are related to ontology modifications.

A number of existing web applications provide query functionalities for specific ontologies like the popular Gene Ontology (GO) (e.g., [10,11]). Furthermore, life science ontologies can be accessed through platforms like BioPortal [12] or OBO Foundry [13]. Although it is possible to retrieve different versions of an ontology, such platforms rarely provide information about evolution, i.e., users have the problem to figure out how an ontology has evolved compared to their version in use. Recently, some web tools offer access to information about the evolution of the Gene Ontology (GO). GOChase [14] allows to study the history of individual GO concepts and Park et al. [15] propose graph-based visualization methods to view modified GO terms. In own previous work we designed the OnEX web application [16] for versioning as well as quantitative and concept-based evolution analysis of life science ontologies. Our tool CODEX [17] can be used to determine a diff between two ontology versions covering complex changes (e.g., concept merge or split). For a general overview on ontology and schema evolution including diff computation we refer to [4]. In summary, currently available tools lack the functionality to analyze and compare evolution in different ontology parts especially for large ontologies with several version releases.

We therefore present the novel web application REX (**R**egion **E**volution e**X**plorer). REX can be used (1) to determine differently changing regions for periodically updated ontologies, and (2) to interactively explore the change intensity of those regions. REX provides a comparative trend analysis such that users and developers can monitor the long-term evolution for their regions of interest, e.g., to track the work or coordinate future development. To show the applicability of REX, we evaluate the tool by analyzing evolution trends in four representative life science ontologies. REX is online available at http://www.izbi.de/rex and provides a web service interface for programmatic access at http://dbs.uni-leipzig.de/wsrex.

This paper is an extended version of [18] presented at DILS 2014. For this version REX has been improved and provides additional features such as the specification of individual cost models and a web service interface for programmatic access. We further describe possible use cases for REX and outline opportunities for future work in more detail. New region evolution analyses have been performed on four representative life science ontologies. The base region discovery algorithm used by REX has been published in [19]. This algorithm allows to detect (un)stable ontology regions for an arbitrary number of ontology versions. However, in this form the algorithm is only applicable offline, i.e., the research community can not make use of it. With the help of REX the algorithm is applicable in two ways: (1) by interactively analyzing region evolution via the web application and (2) by remotely accessing the web service interface. REX fits into our tool suite for ontology evolution management as follows. REX is build upon the OnEX repository [16] offering versioning capabilities for life science ontologies, i.e., ontologies and their versions available in OnEX can be analyzed with REX as well. If someone is interested in detailed changes between two particular ontology versions we refer to the CODEX tool [17] which provides ontology version comparison (diff) facilities.

## Methods

The region discovery method proposed in [19] enables the detection of changing and stable ontology regions. The basic idea is to compute change intensities for regions based on changes between several succeeding versions of an ontology within a specific time interval. First, we briefly describe the applied cost model and region measures. We then describe the region discovery method as well as an algorithm to identify trends in the evolution of ontologies. We present the infrastructure of REX and describe its different workflows and features.

### Region discovery methods

#### Change costs

An ontology consists of a set of concepts which are interrelated by different relationships like *is-a* and *part-of*. Each ontology concept has an unambiguous identifier and is further defined by a set of attributes like its name, synonyms or definition. Discovering changing or stable ontology parts requires the definition of a *cost model* to measure the influence of changes on ontology concepts. In general, ontology content can be added (addition), removed (deletion) or modified (update). Here we distinguish between seven basic change operations for ontology concepts, their attributes and relationships between concepts listed in Table 1. These basic change operations cover all modifications that typically occur in an ontology and are suitable to detect changing ontology regions. More complex change operations (e.g., concept moves) are composed of these basic operations and can be derived by aggregating basic changes to a more compact representation [20]. For instance, a move of a concept within the ontology hierarchy is composed of an addition (*addR*) and a remove (*delR*) of a relationship. Furthermore, typical changes like name and property changes are covered by the change operation *chgAttValue*. Relationship changes with is-a or other semantics (e.g., part-of) are represented by *addR*/*delR*. Our cost model now assigns change costs to each basic change operation, i.e., we can represent the impact of change operations by different costs (see change costs used in REX in Table 1). For instance, we can assign higher costs to deletions since they might have a higher impact on dependent applications than additions. Note, that users can adapt the cost model according to their application scenario. If a user is especially interested in regions that have been heavily extended, she should rank additions higher than deletions. To reflect the impact of changes on concepts, we introduce two types of concept costs: (1) local costs *lc*(*c*) cover the impact of change operations that directly influence a concept *c*, e.g., the change of an attribute value or the addition/deletion of a child concept have a direct impact, and (2) aggregated costs *ac*(*c*) are used to reflect all changes occurring in the *is-a* descendants of a concept *c*, e.g., leaf additions/deletions indirectly influence ancestor concepts. We will later describe how we assign local and aggregated costs to concepts.

**Table 1:** **Change operations and change cost model**

	**Change operation**	**Description**	**Change costs**
Attributes	*addC*	Addition of a new concept	1
	*delC*	Deletion of a concept	2
Relationships	*addR*	Addition of a new relationship	0.5/0.5
	*delR*	Deletion of a relationship	1.0/1.0
Concepts	*addA*	Addition of a new attribute	0.5
	*delA*	Deletion of an attribute	0.5
	*chgAttValue*	Modification/change of an attribute value	0.5

The table shows which change operations and corresponding change costs we utilize in REX. For relationships we split the costs and assign them to the source and target concept, respectively.

#### Regions and measures

**Figure 1:**
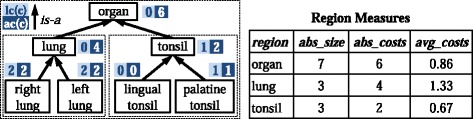
Example part of an anatomy ontology. The figure shows a small yet comprehensive example anatomy ontology to illustrate regions as well as local (*l* *c*(*c*)) and aggregated (*a* *c*(*c*)) costs (left). For instance, the region ‘lung’ consists of three concepts and has aggregated costs of four. The table on the right shows the corresponding results when applying the region measures (*a* *b* *s*_*s* *i* *z* *e*, *a* *b* *s*_*c* *o* *s* *t* *s*, *a* *v* *g*_*c* *o* *s* *t* *s*) in this example.

An ontology region *OR* consists of an ontology concept (region root *rc*) and its *is-a* subgraph, i.e., it covers all leaf and inner concept changes within this region. The definition of our regions covers the experience that changes often occur in the boundary of an ontology, e.g., addition of leaves or subgraphs to extend the knowledge of a specific topic. Of course our regions also cover changes on inner concepts since all intermediate concepts between the root and the leaves are part of the region. As an example Figure 1 (left) illustrates part of an anatomy ontology. We can consider the regions ‘lung’ and ‘tonsil’ each consisting of three concepts. Note that the complete ontology can also be regarded as a region defined by the ontology root ‘organ’.

So far, REX provides a set of measures to describe the change intensity of ontology regions. For each *OR* one can determine its absolute size (*abs*_*size*(*OR*)) w.r.t. the number of concepts. Absolute change costs of an *OR* (*abs*_*costs*(*OR*)) are represented by the aggregated costs of its root *ac*(*rc*). The average change costs per concept in *OR* can be computed as the fraction of absolute change costs and the region size: $avg\_costs(OR) = \frac {abs\_costs(OR)}{abs\_size(OR)}$. Applying these measures to our example results in the values displayed in Figure 1 (right). The ‘lung’ region changed more intensively (*avg*_*costs*(^′^*lung*^′^)≈1.33) compared to ‘tonsil’ (*avg*_*costs*(^′^*tonsil*^′^) ≈0.67). The overall change intensity of the ontology is $\frac {6}{7}\approx 0.86.$

Our general aim is to determine (un)stable ontology regions w.r.t. a specific time interval (*t*_*start*_,*t*_*end*_), i.e., changes between ontology versions released in this interval need to be considered. For this purpose we show first how we can determine local (lc) and aggregated costs (ac) for two versions *O*_*old*_ and *O*_*new*_. Later we will describe how we can generalize the two-version approach for an arbitrary number of versions. For further details about both algorithms we refer to [19]. We will highlight the main steps since the REX application is the main contribution of this article.

#### Region discovery for two versions

The general procedure for two versions is depicted in the following algorithm (computeAggregatedCosts):



The algorithm accepts two versions *O*_*old*_, *O*_*new*_ and a cost model *σ*. Its four main steps are: (1) diff computation, (2) local cost assignment, (3) cost propagation and (4) cost transfer. We first need to determine the difference between both input versions (line 1). For this purpose we can use existing Diff algorithms such as PromptDiff [21] or COntoDiff [20]. The result is the diff *ΔO*_*old*_−*O*_*new*_ consisting of a set of change operations that occurred between *O*_*old*_ and *O*_*new*_.

Using the diff and the change costs *σ* we next assign local costs to concepts which are involved in changes (line 2). Depending on the type of change we assign local costs to concepts in the old or new version. Additions are registered in the new version while deletions are covered in the old version. The assignment further depends on the kind of ontology element that has been changed. Costs from changes on a concept or its attributes are assigned to the concept itself while costs for relationships are split and assigned to the source and target concept of the relationship, respectively.

We now use the two ontology versions annotated with local costs to derive the aggregated costs per concept (line 3-4). In particular, we propagate local costs along is_a paths upwards to the root(s). Due to multi-inheritance we may need to split costs during propagation. The aggregated costs *ac*(*c*) of a concept *c* can be determined as follows:

$$ac(c)=\sum\limits_{c'\in children(c)}{\frac{ac(c')}{|parents(c')|}+lc(c)} $$

The aggregated costs *ac*(*c*^′^) of each child *c*^′^ are divided by the number of parents the child has (|*parents*(*c*^′^)|). These costs are summed up for each child of the considered concept *c* and added to its local costs *lc*(*c*) to finally get its aggregated costs *ac*(*c*). We thus distribute costs in the case of multiple inheritance and finally ensure that the root concept(s) of the ontology contain the overall sum of all assigned local costs. In our example in Figure 1 (left) the aggregated costs of ‘organ’ (*ac*(^′^*organ*^′^)=6) are computed based on the aggregated costs of its children *ac*(^′^*lung*^′^)=4 and *ac*(^′^*tonsil*^′^)=2 as well as its own local costs *lc*(^′^*organ*^′^)=0.

In order to determine (un)stable regions in the new version, we need to transfer costs from *O*_*old*_ into *O*_*new*_ (line 5). We therefore sum up aggregated costs which belong to the same concept in the old/new version. After this step we can apply our region measures as defined earlier or use the new ontology version with aggregated costs for further processing (see Multiple Version algorithm).

#### Region discovery for multiple versions

We generalize our basic algorithm for multiple released versions *O*_1_, …, *O*_*n*_ by executing it *n*−1 times so that we successively determine aggregated costs (for each version change *O*_*i*−1_↦*O*_*i*_) and transfer them to the newest version *O*_*n*_. In *O*_*n*_ we can apply the previously described region measures. The overall algorithm findRegions looks as follows:



#### Trend discovery for regions

Using the region discovery method (findRegions) one can determine the most (un)stable regions for a specific time interval. To better monitor region changes over long periods of time and to figure out trends in their evolution, we propose a further method for trend discovery based on sliding windows. The overall procedure trendDiscovery looks as follows: Using the region discovery method (findRegions) one can determine the most (un)stable regions for a specific time interval. To better monitor region changes over long periods of time and to figure out trends in their evolution, we propose a further method for trend discovery based on sliding windows. The overall procedure trendDiscovery looks as follows:



The algorithm works on an ontology *O*, a time interval (*t*_*start*_, *t*_*end*_) and an ontology region of interest *OR* to be monitored. We further use a sliding window of size *ω*, a step width *Δ* and change costs *σ*. In particular, we successively shift the window beginning at *t*_*start*_−*ω* over the time interval until we reach its end *t*_*end*_. In each step we first determine the released ontology versions within the window (line 3). We then calculate and save the costs (e.g., *avg*_*costs*) for *OR* by calling the region discovery algorithm (discoverRegions) for the versions within *ω*. We thus generate a time-based map (line 6) containing information about the change intensity of *OR* at specific points in time in the defined window. The results are visualized for users in the *Trend Analysis* component of REX.

### Web application

#### Architectural overview

**Figure 2:**
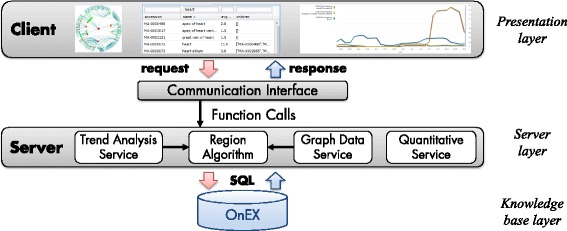
Three-layered architecture of REX. The figure shows the architecture of REX consisting of three layers: (1) knowledge base layer, (2) server layer, (3) presentation layer.

REX is based on a three-layered architecture displayed in Figure 2. The back-end consists of the OnEX repository [16] which currently provides access to more than 1,000 versions of 16 popular life science ontologies. Note that it supports the import of ontologies in different formats such as OWL and OBO. Users can analyze integrated versions with the offered facilities of REX. The server layer is implemented in Java and realizes different services to access ontology versions in OnEX. Moreover, it provides services to calculate the region measures and to perform trend and quantitative analyses. Every service is encapsulated in its own module, such that it is possible to change the region discovery algorithm independently of the other modules. Results are transformed such that the application can visualize ontologies and changing regions in graphs. Moreover, we provide a web service for programmatic access. So far, it computes the average costs per concept for a particular ontology and time interval. Ontology developers are thus able to integrate REX functionalities into their own applications. For instance, a set of annotations could be automatically rejected, if the average costs of involved concepts exceed a given threshold. The front-end is a platform-independent web application based on the Google Web Toolkit (GWT)[22] and the graph library InfoVis[23]. In the following we discuss the analysis facilities of REX, namely the *Structural Analysis*, *Quantitative Change Analysis* and *Trend Analysis*, as well as the web service interface, in more detail.

#### Structural analysis

**Figure 3:**
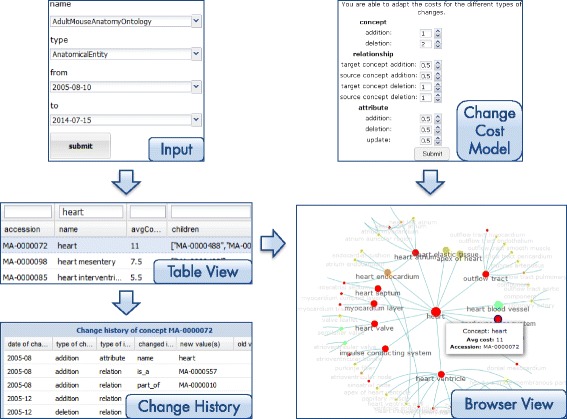
Structural Analysis component. The figure shows the structural analysis component of REX.

The structural analysis component represents the evolution of regions in an ontology for a specified time interval as a graph (Figure 3). The component is mainly divided into a *Browser View* as well as a table to search and filter results (*Table View*). First the user needs to specify the ontology name and the time period to review in the *Input* form. Moreover, users can adapt the applied change cost model according to their analysis szenario (*Change Cost Model*). The system then performs the region discovery algorithms and generates a graph to visualize the results (*Browser View*). Each node in the graph represents an ontology concept, *is-a* relationships are displayed as edges between the nodes. The layout is circular and displays a concept and its near neighborhood, i.e., its descendants and parent nodes (either with or without labels). Users can easily identify interesting sub regions by selecting a concept in the graph (*Browser View*) or in the *Table View*. This concept is then shown as the central node in the *Browser View*. It is possible to navigate in both directions through the ontology. For instance, if one is interested in a specific sub region and its content, one clicks on the node and the graph will display the sub region in more detail. In contrast, one can also navigate to a more general concept (surrounded by blue circles) to see sibling regions of the current one. The colors signal the measured change intensity (*avg*_*costs*) of a region. Red stays for high change intensity whereby green is used to mark stable regions. Thus, users can easily figure out where (un)stable regions are located. We provide two coloring schemes: (1) interval-based grouping or (2) equal distribution between min/max *avg*_*costs*. For each concept in the graph, small info boxes (’mouse over’) provide further information like the accession number, concept name/label or the measured *avg*_*costs*.

In general the number of concepts and relationships in an ontology is very high. Thus, it is difficult to recognize interesting regions only by browsing through the graph especially for large ontologies. Moreover, users may be interested in the change intensity of specific regions. The *Table View* therefore allows users to filter and sort ontology regions by their accession number, name and *avg*_*costs*. In particular, search criteria can be specified in the head of the table to find regions of interest. For instance, one can filter out all regions in the Adult Mouse Anatomy Ontology containing the name ‘heart’. Users can simply select their region of interest in the table and move to the *Browser View* for its visualization. To get a more detailed view of occurred changes, users can request the local *Change History* of a selected concept at the bottom of the table.

#### Quantitative change analysis

**Figure 4:**
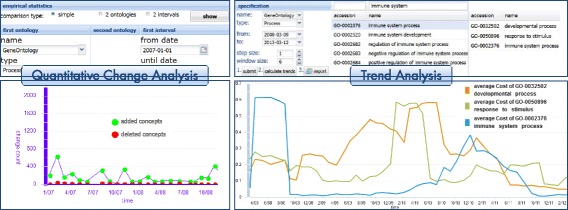
Quantitative Change and Trend Analysis components. The figure shows the quantitative change and trend analysis components of REX.

To get information about how many changes occurred in an ontology for a specific time interval REX offers the quantitative change analysis component (Figure 4 left). Users can generate diagrams to see the differences between released ontology versions in statistical (quantitative) form, i.e., we count and visualize how many changes (*addC*, *delC*, *addR*, *delR*) occurred. In particular, users can display the number of changes in one ontology for a specific time interval, e.g., GO Biological Processes in 2013. Moreover, one can compare the evolution of two different ontologies for a specified time interval or compare two different time intervals for the same ontology. Users can thus identify interesting ontologies and time periods for later region analyses.

#### Trend analysis

The trend analysis component can be used to study and compare the long-term evolution of selected regions (Figure 4 right). Users first need to specify the ontology, the time interval (first and last version) and the window size and step width (number of versions). Next they are able to select regions of their interest either by searching the respective accession number/concept name or by choosing from top-level concepts of the ontology. REX executes the proposed trendDiscovery algorithm to measure the *avg*_*costs* for the selected regions at different points in time. The results are converted into a line chart which displays the trend of the measured *avg*_*costs* for each region over time. Users are thus able to compare the change intensity for different regions of interest within one diagram.

#### Web service

In addition to the web application, we provide a JAX web service for programmatic access to REX. The web service interface is available at http://dbs.uni-leipzig.de/wsrex?wsdl. Programmers can apply the region discovery methods for a specified ontology and a defined time interval. Using the provided WSDL description they can generate the corresponding client classes to enable web service interaction. We provide three methods building on each other:

- *getAvailableOntologies* returns all existing ontologies in our OnEX repository.
- getVersions returns a list of available versions for a specified ontology.
- *calculateRegions* calculates the average costs for each concept in the specified ontology and time interval. It returns a list of concepts including accession numbers, concept names and the computed average costs for each concept.

## Results and discussion

In the following we will describe and discuss some selected results generated with REX. In particular, we will present results for the following well-known life science ontologies: Gene Ontology (GO) with its sub ontologies Molecular Functions (GO-MF), Biological Processes (GO-BP) and Cellular Components (GO-CC), the Thesaurus of the National Cancer Institute (NCIT), Adult Mouse Anatomy ontology (MA) and Chemical Entities of Biomedical Interest (ChEBI). We will focus on results for the recent past (mainly 2012-2013). Note that users can flexibly use REX to explore evolution trends for regions in other available ontologies for arbitrary time intervals. We first discuss the evolution in general (quantitative statistics) and show the change intensities for whole ontologies. We then describe the usage of the structural analysis and trend analysis components of REX by different examples.

### Evolution in general

Usually, the evolution of an ontology can be described by the number of basic changes (e.g, *addC*, *delC*, *addR*,*delR*) occurred. For a start, the quantity of change operations provides an indication of how an ontology evolved, e.g., an ontology exhibiting a small number of changes over the time can be classified as stable. However, the location, i.e., information about the region where changes occurred is missing. Table 2 shows the quantity of additions and deletions of concepts and relationships for the considered ontologies in 2012 and 2013 generated with the quantitative change analysis component of REX. Overall, every ontology has been modified in the considered time intervals. An exception forms MA, where no (only one) version was released in 2012 (2013). In general the ontologies grow, i.e., the quantity of insertions (*add*) is higher than the quantity of deletions (*del*). Most changes occurred in NCIT and ChEBI, e.g., more than 12,000 concepts have been added in both ontologies. However, there has also been an increased number of deletions, i.e., the ontologies were optimized by rearranging concepts in the hierarchy or by merging multiple redundant concepts into a single one.

**Table 2:** **Quantitative analysis results**

	**2012**				**2013**			
	***addC***	***delC***	***addR***	***delR***	***addC***	***delC***	***addR***	***delR***
GO-BP	2,914	51	11,940	2,844	1,159	91	5,742	2,812
GO-MF	461	62	1,159	379	126	6	431	179
GO-CC	185	3	581	124	219	4	597	341
ChEBI	7,961	60	15,803	1,713	4,323	70	17,010	2,830
NCIT	4,878	109	6,064	1,115	8,327	174	9,183	958
MA	-	-	-	-	-	-	-	-

The table shows the quantity of changes occurred in the ontologies under investigation. We distinguish between *addC*, *delC*, *addR* and *delR* changes for two periods namely 2012 and 2013. We considered available versions (at least two) within a period. MA has released no (only one) version in 2012 (2013). Thus, no statistics are provided for MA.

We apply our region algorithm to measure the change intensity of whole ontologies. In particular, we use the root concept(s) of an ontology as regions, i.e., we aggregate all costs in the root(s) and can thus estimate the overall ontology change intensity for a specific time interval. Additional file 1: Table S1 displays the change intensities (*abs*_*size*, *abs*_*costs*, *avg*_*costs*) for all ontologies under investigation in 2012 and 2013. The ontologies show different behaviors in their change intensities. In both periods ChEBI exhibits the highest absolute costs. Its change intensity even increased from 2012 compared to 2013 (*avg*_*costs*: 0.88 ↦0.95). Similarly, other ontologies like GO-CC or NCIT have been modified more extensively in 2013. In contrast, the GO sub ontologies GO-BP and GO-MF show decreased change intensities in 2013 compared to 2012, i.e., modification actions on these ontologies have been reduced. Regarding GO, GO-BP is the sub ontology with the most frequent changes in both years. MA is relatively stable since only slight changes occurred in 2013.

### Structural analysis

**Figure 5:**
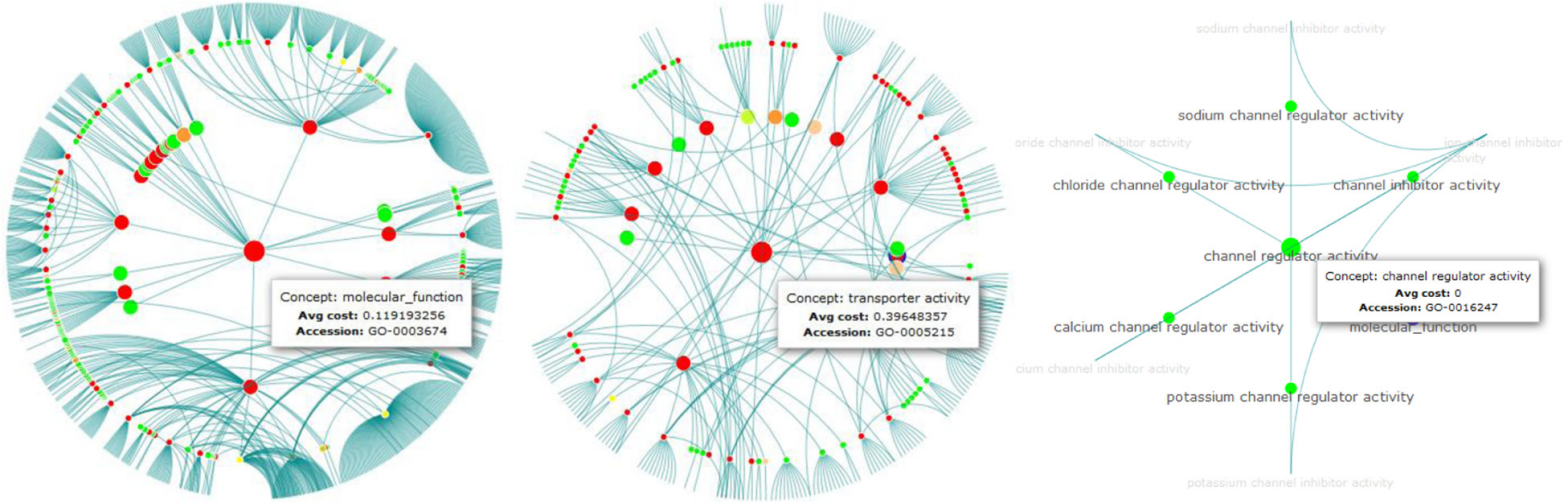
Structural analysis for GO-MF in 2013. The figure shows the sub graphs of the root concept GO:0003674 ‘molecular_function’ (left), GO:0005215 ‘transporter activity’ (middle) and GO:0016247 ‘channel regulator activity’ (right). Measured change intensities (*a* *v* *g*_*c* *o* *s* *t* *s*) are displayed using a red-green scale (green: stable, i.e., less *a* *v* *g*_*c* *o* *s* *t* *s*; red: unstable, i.e., increased *a* *v* *g*_*c* *o* *s* *t* *s*).

After focusing on the overall ontology change intensity, we will now show how one can use the structural analysis component to explore details about the evolution in different regions of an ontology. We describe the usage of the structural analysis component for GO-MF in 2013. GO-MF has two parts namely ‘molecular_function’ (GO:0003674) which contains all active molecular functions and ‘obsolete_molecular_function’ (GO:0008369) used to collect all obsolete (inactive) concepts. All main regions are direct children of GO:0003674. The browser view shows, that the majority of these regions are unstable (see red nodes next to the central node in Figure 5 left). For instance, ‘transporter activity’ (GO:0005215) has *avg*_*costs* of 0.4 which are greater than those of ‘molecular_function’ (0.12). Furthermore, many children (sub regions) of ‘transporter activity’ show high *avg*_*costs* (Figure 5 middle). This indicates that the whole region of ‘transporter activity’ has significantly changed compared to other regions in 2013 that show low *avg*_*costs* since less or even zero changes occurred. For instance, the ‘channel regulator activity’(GO:0016247) region has *avg*_*costs* of zero, i.e., no concept in this region has been modified in 2013 (Figure 5 right).

**Figure 6:**
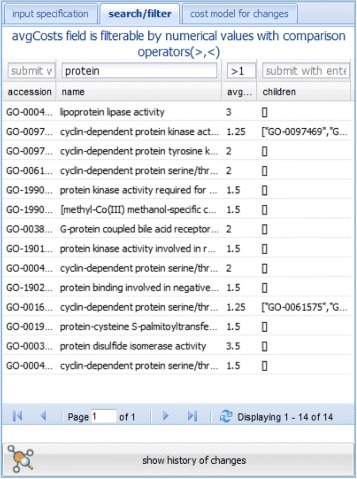
Specification of a filter on the name column and *a* *v* *g*_*c* *o* *s* *t* *s* in the table view. The figure shows the specification of a filter on the name column for GO-MF in 2013. In particular, we search for all regions related to ‘protein’ having *a* *v* *g*_*c* *o* *s* *t* *s*>1. For GO-MF in 2013 14 regions satisfy this criteria.

Instead of browsing, one can use the table view to locate interesting regions by specifying different filter criteria. For instance, to select all regions in GO-MF related to the term ‘protein’, one can specify a filter condition on the name column (Figure 6). REX selects and displays all regions that satisfy this criteria, e.g., for GO-MF in 2013 we find 557 regions related to ‘protein’. Users can further specify conditions on *avg*_*costs* to find strongly changing or stable regions. In our case we may look for regions related to ‘protein’ having *avg*_*costs*>1, i.e., we search for unstable regions related to ‘protein’ (Figure 6). We can thus reduce the selection from 557 to 14 regions satisfying both criteria. Based on this selection (and a possible sorting) users can now select a region of interest to create a corresponding graph in the browser view for a more detailed inspection.

**Figure 7:**
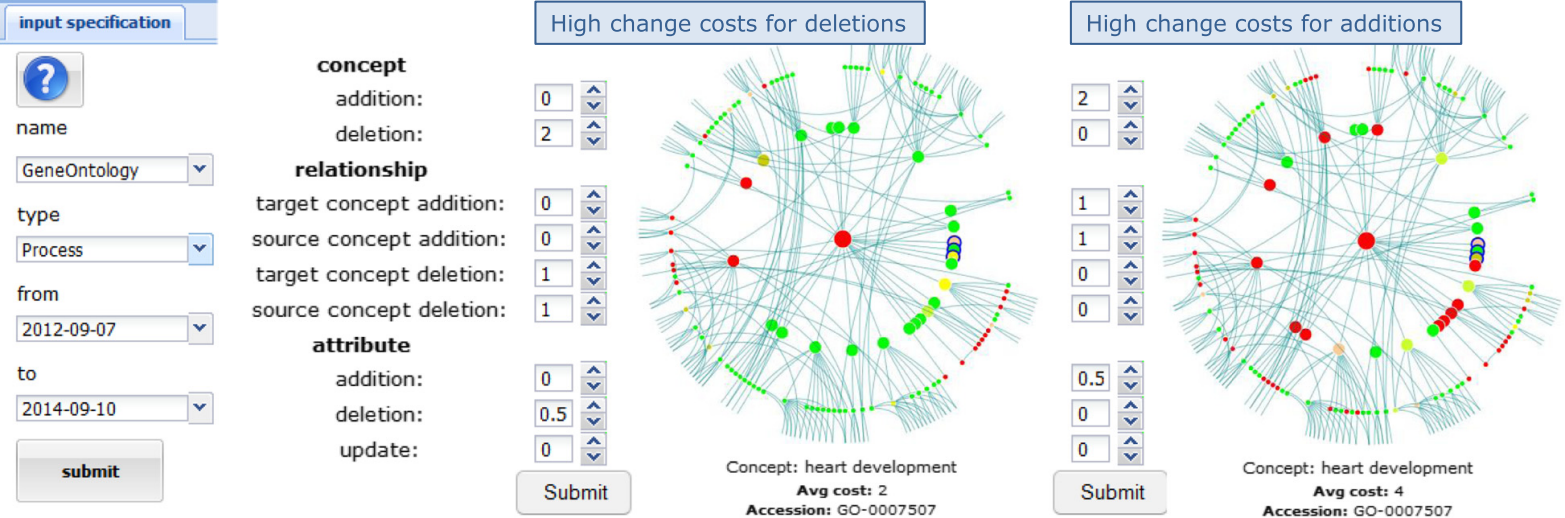
Application of different cost models. The figure shows results for the application of different cost model specifications for the concept ‘heart development’ in GO-BP between 09-2012 and 09-2014. To visualize the impact of different cost models, we assign high costs to deletions (left) and additions (right), respectively. Red nodes on the left (right) denote regions where predominantly deletions (additions) took place.

We further allow to modify the applied cost model. Dependent on the application scenario users might be mainly interested in one/some of the used change operations (e.g., *addC*, *addR*, …), i.e., they should rank the respective costs higher. One user might like to know which ontology parts were of high research interest and have been strongly extended in the near past (many additions). Another user might be looking for regions where many deletions took place since she needs to know whether her application is affected by many information reducing changes (many deletions). To visualize the impact of different cost models, we exemplary assign high costs to deletions (*delC*, *delR*, *delA*) and additions (*addC*, *addR*, *addA*), respectively. Figure 7 shows results for the concept ‘heart development’ in GO-BP between September 2012 and 2014. Red nodes on left (right) denote regions where predominantly deletions (additions) took place. The results show that a variation of the cost model impacts the computation of stable or unstable regions. Many subregions of ‘heart development’ have been mainly extended (red nodes on the right side) whereas only two subregions where affected by a high number of deletions (red nodes on the left side).

### Trend analysis

**Figure 8:**
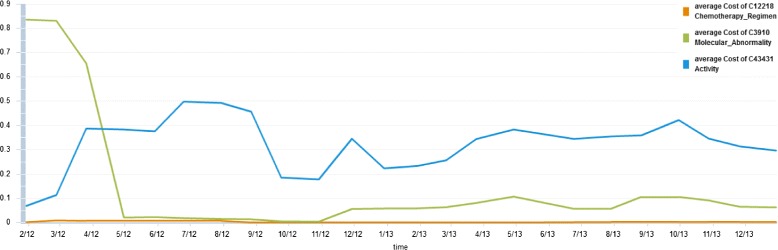
Trend analysis for selected regions of NCIT between 2012-2013. We perform a trend analysis for three regions of NCIT between 2012-2013: ‘Chemotherapy_Regimen’ (C12218), ‘Molecular_Abnormality’ (C3910) and ‘Activity’ (C43431). The figure shows how their change intensity (*a* *v* *g*_*c* *o* *s* *t* *s*) evolved over time when using a sliding window of length six months and a step width of one month.

As an example, we will show results for a two-year trend analysis in NCIT between 2012 and 2013. In particular, we select the three regions ‘Chemotherapy_Regimen’ (C12218), ‘Molecular_Abnormality’ (C3910) and ‘Activity’ (C43431) and measure their change intensity (*avg*_*costs*). We choose a sliding window of six versions (window size *ω*) and shift the window by one version in each step (step width *Δ*). Figure 8 displays the generated result chart. The three regions show a different behavior in their change intensity. The work on ‘Molecular_Abnormality’ was mainly performed in the beginning of 2012 (*avg*_*costs* up to 0.9) before its change intensity decreased to nearly zero, i.e., one might consider this region as one that became stable over time. The ‘Chemotherapy_Regimen’ (C12218) region was stable in the complete period (*avg*_*costs*<0.05), i.e., the development in this region was probably performed before 2013 and it seems that the region will be stable in the near future as well. On the other hand, such a long-term stable region might have just been of low interest in the past and needs future development. In contrast, the region on ‘Activity’ (C43431) has been continuously adapted during the whole analysis period. It seems to be of high research interest and is still under development such that it is likely to be further changed in the next months or years. Users that are especially interested in content of this region for their analyses/workflows need to take care of the ongoing evolution. In contrast those working within the ‘Molecular_Abnormality’ and ‘Chemotherapy_Regimen’ regions can assume that their regions of interest will be relatively stable in the near future. The trend analysis of REX is valuable to support ontology development since the evolution of ontologies can be monitored over longer periods in time. Of course, the interpretation of trend results is up to the user and depends on their specific application scenario.

## Conclusions and future work

REX provides interactive access to information about the evolution of life science ontologies. Users can explore (un)stable ontology regions by different workflows. The knowledge about changing ontology regions can be used to support ontology-based algorithms and analysis. Furthermore, the development of large life science ontologies can be monitored with REX, i.e., developers and project coordinators can inform themselves about ongoing work in different ontology parts.

For future work, we plan to extend REX such that users are able to perform region analysis on their individual ontologies. We will further extend the change cost computation of REX by involving alternative metrics for changing concepts. For instance, we can involve semantic similarities or distances between ontology concepts (see [24] for an overview) to include the near context of a changed concept, i.e. changes on ancestor as well as descendant concepts. Effects of “dense” local changes might have more impact, and could by ranked higher during change intensity computation. Moreover, we like to perform a more detailed evaluation with ontology developers to analyze how REX can be used in ontology development and application scenarios. In [9] we already used the Region Discovery Algorithm to analyze Gene Ontology changes in the context of the widely used term enrichment analyses. It would be further interesting to see if specific evolution trends are in accordance with editorial policies or specific activities in sub-domains. It might be helpful to provide a suitable presentation of REX results, e.g., by integrating its functionalities into tools used by the ontology developers or annotation curators. Currently, the GOA consortium uses the tool *Protein2GO* for annotation and emphasizes curation and quality control of GO annotations [25]. So far, it does not involve information on ontology evolution. Curators could be supported by presenting REX’ change intensities for newly created and existing annotations to indicate whether further quality control might be necessary, e.g., due to significant changes in the considered ontology part. To better support the ontology development process with information about the evolution in different ontology regions, we like to provide REX plugins for common tools like Protégé [26] or OBO-Edit [27]. The plugins should be able to flexibly present ontologies and their changing regions. For instance, developers might prefer a reduced presentation of the hierarchies, e.g., by focusing on highly changing regions that cover frequently used concepts or by dividing concepts of an ontology into smaller, more manageable units [28].

## Additional file

Additional file 1
**Table S1.** Change intensity of complete ontologies in 2012 and 2013. The table shows the change intensity for each ontology under investigation in 2012 and 2013. The three columns per year display the ontology size (*a* *b* *s*_*s* *i* *z* *e*) and the measured absolute costs (*a* *b* *s*_*c* *o* *s* *t* *s*) as well as average costs (*a* *v* *g*_*c* *o* *s* *t* *s*). The red-green scale for *a* *v* *g*_*c* *o* *s* *t* *s* highlights ontologies with high (red) and low (green) change costs. We performed the region discovery algorithm for released versions in 2012 and 2013, and considered the root concept(s) as region(s). For ontologies with multiple root concepts we summed up the absolute costs per root concept and calculated the average costs w.r.t. the overall ontology size.
