# Supplementary material for: Region Evolution eXplorer – A tool for discovering evolution trends in ontology regions
Source: J Biomed Semantics. 2015 Jun 1;6:26. doi: 10.1186/s13326-015-0020-6 (PMC4450457; doi:10.1186/s13326-015-0020-6)
Supplement: Additional file 1 — Table S1. Change intensity of complete ontologies in 2012 and 2013. The table shows the change intensity for each ontology under investigation in 2012 and 2013. The three columns per year display the ontology size (a b s_s i z e) and the measured absolute costs (a b s_c o s t s) as well as average costs (a v g_c o s t s). The red-green scale for a v g_c o s t s highlights ontologies with high (red) and low (green) change costs. We performed the region discovery algorithm for released versions in 2012 and 2013, and considered the root concept(s) as region(s). For ontologies with multiple root concepts we summed up the absolute costs per root concept and calculated the average costs w.r.t. the overall ontology size. [file 13326_2015_20_MOESM1_ESM.pdf]

	**2012**			**2013**		
	abs_size	***abs_costs***	***avg_costs***	***abs_size***	***abs_costs***	***avg_costs***
**GO-BP**	25,021	22,278	0.89	26,133	15,440	0.59
**GO-MF**	10,416	3,158	0.30	10,546	1,164	0.11
**GO-CC**	3,225	1,153	0.36	3,464	2,080	0.60
**ChEBI**	38,360	33,615	0.88	42,946	40,894	0.95
**NCIT**	95,949	21,000	0.22	105,111	29,970	0.29
**MA**	3,113	0	0.00	3,174	187	0.06
